# Ilex Guayusa Tea Improves Glycaemia and Autonomic Modulation in Female Streptozotocin-Induced Diabetic Rats

**DOI:** 10.3390/ph18030316

**Published:** 2025-02-24

**Authors:** Tafne Coelho Mello, Danielle da Silva Dias, Nathalia Bernardes, Amanda Aparecida do Araujo, Camila Paixão dos Santos, Susana Llesuy, Kátia De Angelis, Filipe F. Stoyell-Conti

**Affiliations:** 1 Laboratory of Translational Physiology, Universidade Nove de Julho (UNINOVE), São Paulo 01504-001, Brazil; tafnec.mello@gmail.com (T.C.M.); danielledias@outlook.com (D.d.S.D.); nbernardes@outlook.com (N.B.); amanda.personal@hotmail.com (A.A.d.A.); camilapaixao22@hotmail.com (C.P.d.S.); 2 Department of Physiology, Federal University of Sao Paulo (UNIFESP), São Paulo 04021-001, Brazil; 3 Postgraduate Program in Physical Education, Universidade Federal do Maranhao (UFMA), Sao Luis 65085-580, Brazil; 4 Postgraduate Program in Physical Education, São Judas Tadeu University, São Paulo 05503-001, Brazil; 5 Instituto Universitario Hospital Italiano, Hospital Italiano de Buenos Aires, Buenos Aires C1199, Argentina; susanallesuy46@hotmail.com; 6 Department of Surgery, University of Miami, Coral Gables, FL 33146, USA

**Keywords:** ilex guayusa, diabetes, cardiovascular autonomic modulation, oxidative stress

## Abstract

**Background**: Cardiovascular diseases are the leading cause of morbidity and mortality among diabetic patients, with their incidence rising globally. Streptozotocin (STZ)-induced diabetic rats, untreated with insulin, exhibit human-like symptoms such as hyperglycemia, polydipsia, polyuria, weight loss, cardiomyopathy, neuropathy, and oxidative stress. Thus, this study evaluated the effects of Ilex guayusa tea on cardiovascular, autonomic, metabolic, and oxidative stress parameters in diabetic rats, as well as its antioxidant and phytochemical properties. **Methods**: Thirteen female Wistar rats were divided into two groups: diabetic (D; *n* = 7) and diabetic + Ilex guayusa (DG; *n* = 6). Diabetes was induced by a single STZ injection (50 mg/kg, i.v.), and guayusa tea was provided ad libitum for 21 days (3.0 g/L). **Results**: Glycemia and body mass were initially similar between the groups; however, throughout the protocol, the D group showed an increase in glycaemia and a decrease in body mass when compared to initial values. While no differences in arterial pressure, heart rate, linear heart rate variability, and oxidative stress were observed, the D group showed reduced vascular sympathetic modulation (LF-SAP and VAR-SAP) compared to the DG group. This reduced vascular sympathetic modulation, which was a poor prognostic marker in this model, was inversely correlated with glycemia (VAR-SAP and final glycemia, r: −0.81, *p* = 0.002). **Conclusions**: These findings suggest that Ilex guayusa tea consumption may mitigate metabolic and autonomic dysfunction in diabetes, highlighting its potential therapeutic role in managing diabetic complications.

## 1. Introduction

The prevalence of diabetes mellitus (DM) is rising dramatically worldwide, creating a significant global health challenge [1]. A strong association exists between DM and cardiovascular disease (CVD), the leading cause of morbidity and mortality in diabetic patients [2]. Adult men with DM face a 1.7-fold higher CVD mortality rate than nondiabetic individuals [3]. It is important to highlight that women with DM experience even higher relative risks (2.2-fold) for CVD compared to men [3,4]. The multifaceted nature of DM-CVD interaction includes obesity, hypertension, dyslipidemia, oxidative stress, endothelial dysfunction, and autonomic neuropathy, all of which exacerbate CVD risks.

Given the rising prevalence and complexity of DM, reducing CVD outcomes should be a primary focus of diabetes management. Epidemiological studies and meta-analyses consistently suggest that tea consumption offers protective effects against cardiovascular disease. The potential mechanisms underlying these benefits include tea’s ability to lower blood pressure, improve blood lipid profiles, regulate glucose levels, and support healthy body weight management [5]. Although several different teas have been studied regarding their effects on cardiometabolic parameters, few studies verified the proprieties and effects of Ilex guayusa.

Guayusa (Aquifoliaceae Ilex guayusa) is a holly tree found at elevations up to 2000 m in the countries of Ecuador, Colombia, and Peru. The Kichwa tribes of Napo Ecuador have chewed and consumed outright guayusa leaves and/or have dried and brewed them like a tea for their stimulative effects. Kapp et al. [6], after examining the in vitro and in vivo general toxicity data and determining the safety of the use of a standardized liquid concentrate of guayusa derived from guayusa leaves that had been boiled in water, observed no evidence of any general or genetic toxicity nor any evidence of any concern to consumers of guayusa tea. Moreover, Pardau et al., in 2017, demonstrated that Ilex guayusa teas are, in fact, a good source of dietary phenolic compounds with cellular antioxidants and anti-inflammatory properties [7].

Interestingly, rats induced with diabetes by streptozotocin (STZ) and untreated with insulin show similar alterations seen in humans, such as hyperglycemia, polydipsia, polyuria, and weight loss, as well as cardiomyopathy, diabetic neuropathy, and increased oxidative stress [8]. Therefore, in this study, we assessed the effects of Ilex guayusa tea on cardiovascular, autonomic, metabolic, and oxidative stress parameters of female STZ-induced diabetic rats, as well as its antioxidant and phytochemical properties.

## 2. Results

### 2.1. Antioxidant and Phytochemical Properties of Ilex Guayusa

To confirm the strong antioxidant properties of Ilex guayusa, in vitro antioxidant activity assays (TRAP, ABTS, DPPH, and RP) were conducted (Table 1). The ABTS assay revealed values 2.2-fold higher for the decoction compared to the infusion. Similarly, the decoction demonstrated superior performance in the DPPH and RP assays, with values 2.1-fold higher than the infusion for both assays. In the TRAP assay, the decoction and infusion presented similar values.

The total polyphenolic and flavonoid contents are presented in Table 2. The decoction showed a significantly higher polyphenol and flavonoid content compared to the infusion.

### 2.2. Metabolic Evaluations

**Figure 1:**
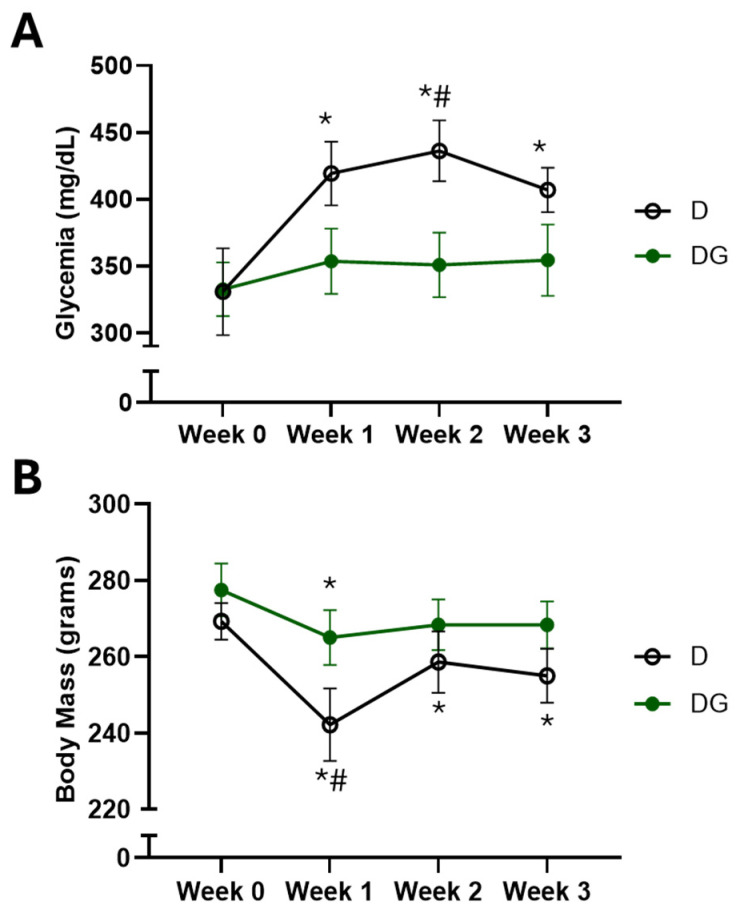
Metabolic assessment in the D (*n* = 7) and DG (*n* = 6) groups. (**A**) Glucose and (**B**) body mass measured before starting Ilex guayusa tea consumption (Week 0) and once a week over three weeks after starting Ilex guayusa tea consumption. * *p* < 0.05 vs. initial values; # *p* < 0.05 vs. DG group.

After confirming the high polyphenolic and flavonoid contents in the Ilex guayusa tea, which present a well-established hypoglycemic effect, we decided to verify the effects of Ilex guayusa tea consumption on STZ-induced diabetic rats. It is important to mention that there was no difference between the groups regarding water/tea (D: 63.6 ± 6.4 vs. DG: 71.4 ± 3.9 mL/day/animal) and food (D: 26.4 ± 3.1 vs. DG: 27.6 ± 5.1 g/day/animal) consumption. Interestingly, we observed that although it did not reduce hyperglycemia development in these animals, the guayusa tea was able to delay it (Figure 1A). In addition, at the end of the protocol, the DG group was able to maintain its body mass in relation to its initial values, while the D group had a significant reduction in body mass (Figure 1B). Finally, no differences were observed between the groups for triglycerides at the end of the protocol (D: 189 ± 26 vs. DG: 126 ± 18 mg/dL).

### 2.3. Hemodynamic and Autonomic Assessments

**Figure 2:**
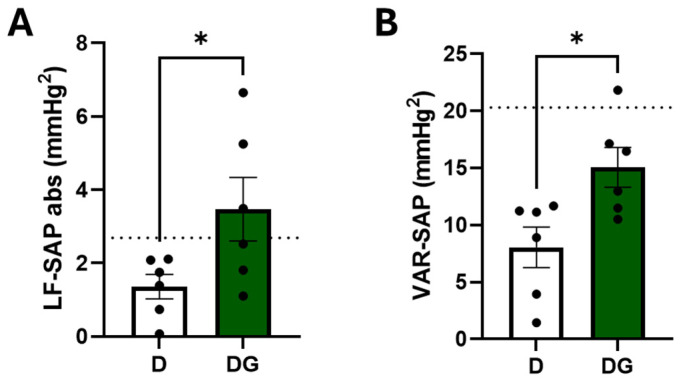
Vascular autonomic assessment in the D (*n* = 6) and DG (*n* = 6) groups. (**A**) Absolute values from the low-frequency band of systolic arterial pressure (LF-SAP); (**B**) variance in systolic arterial pressure at the end of the protocol. Dotted lines represent the average values from three different studies in nondiabetic rats [9,10,11]. * *p* < 0.05.

**Figure 3:**
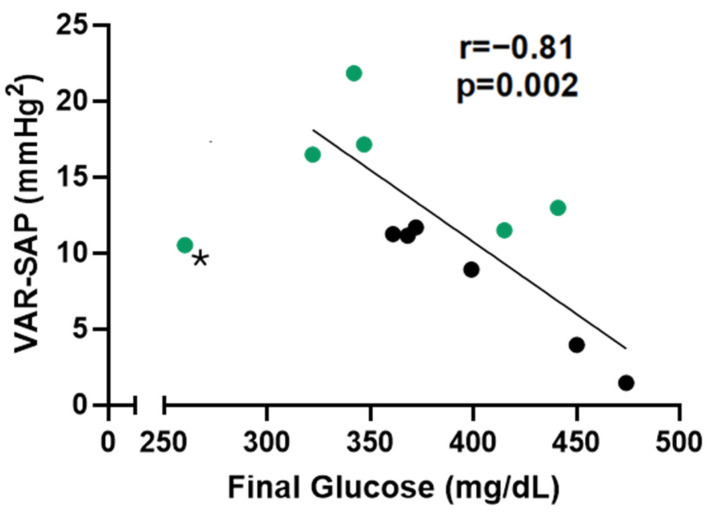
Correlations between final glucose and VAR-SAP. * Outlier (Cook’s distance: 1.87) removed from the analysis. When the outlier was kept in the analysis, the values were: r = −0.60 and *p* = 0.04. Black dots: D group. Green dots: DG group.

STZ-induced diabetic rats, as seen in humans, present diabetic neuropathy, which affects hemodynamic and autonomic control. In this study, we did not observe any differences regarding hemodynamic parameters (MAP of HR) nor in the linear analysis of HRV (Table 3). However, the STZ-induced diabetic rats treated with guayusa for 3 weeks showed higher values of alpha 1 (Table 3), a low frequency of systolic arterial pressure (LF-SAP), and variance in systolic arterial pressure (VAR-SAP) (Figure 2A,B). Moreover, we observed an inverse correlation between final glucose and VAR-SAP (r = 0.81, *p* = 0.002) (Figure 3).

### 2.4. Brain and Cardiac Oxidative Stress of STZ-Induced Diabetic Rats Treated with Guayusa Tea

Given that Ilex guayusa is a rich source of dietary phenolic compounds with cellular antioxidant and anti-inflammatory properties [7], we next evaluated the oxidative profile of the brain and cardiac tissues in STZ-induced diabetic rats treated with guayusa tea. However, we did not observe any differences in all parameters evaluated between the D and DG groups (Table 4).

## 3. Discussion

In this study, we assessed the antioxidant and phytochemical profile of Ilex guayusa tea, as well as the cardiovascular and metabolic effects of its consumption in female STZ-induced diabetic rats. In fact, we were able to confirm its high antioxidant properties and demonstrate the beneficial effects of Ilex guayusa tea consumption on glycemic control and autonomic modulation in these animals.

As demonstrated by others, we also observed high antioxidant properties of *Ilex guayusa* tea, measured by four different methods: TRAP, ABTS, DPPH, and RP. These findings align with previous studies that highlight the significant antioxidant capacity of *Ilex guayusa*, attributed to its rich polyphenolic and flavonoid content [7,12]. Garcia-Ruiz et al. [12] identified a total of 14 phenolic compounds in *Ilex guayusa*, with chlorogenic acid and quercetin-3-O-hexose being the main representatives of hydroxycinnamic acids and flavonols, respectively. Additionally, lutein, the most abundant carotenoid in *Ilex guayusa*, contributes to its antioxidant properties [13].

Interestingly, flavonoids, such as quercetin-3-O-hexose, exhibit potent antidiabetic properties by modulating key processes involved in carbohydrate digestion, insulin signaling, secretion, glucose uptake, and adipose tissue deposition [14]. These compounds influence multiple molecular targets, regulating critical pathways that enhance β-cell proliferation, stimulate insulin secretion, reduce β-cell apoptosis, and alleviate hyperglycemia by optimizing glucose metabolism in the liver [15]. Chlorogenic acid, another major compound in *Ilex guayusa*, has been shown to improve insulin sensitivity and reduce blood glucose levels by inhibiting glucose-6-phosphatase, an enzyme involved in hepatic glucose production [16]. These mechanisms suggest that the polyphenolic and flavonoid content of *Ilex guayusa* may play a significant role in its antidiabetic effects.

To further explore these effects, we investigated the impact of *Ilex guayusa* consumption on glucose levels in female STZ-induced diabetic rats. Although the decoction method demonstrated superior in vitro antioxidant activity and higher total phenolic and flavonoid contents compared to the infusion, we chose the infusion method for its practicality and feasibility in real-world applications. At baseline, both experimental groups exhibited similar levels of hyperglycemia. However, over the course of the study, the diabetic control group (D) experienced a progressive increase in glycemia, which was mitigated in the *Ilex guayusa*-treated group (DG). This hypoglycemic effect in the DG group may be attributed to the high polyphenol and flavonoid content of *Ilex guayusa* tea, consistent with prior findings by Swanston-Flatt et al. [17], who demonstrated that *Ilex guayusa* delayed hyperglycemia development in STZ-induced diabetic rats while also reducing hyperphagia, polydipsia, weight loss, and glycated hemoglobin levels.

Moreover, triterpenoids such as oleanolic acid and ursolic acid, which have been isolated from *Ilex guayusa* [18], are known to enhance glucose tolerance and insulin secretion, further supporting its antidiabetic potential [19,20]. These compounds, along with the antioxidant and anti-inflammatory properties of *Ilex guayusa*, contribute to its multifaceted role in diabetes management.

Given the strong association between DM and CVD, we next evaluated the hemodynamic and cardiovascular autonomic modulation parameters of these animals. No differences in arterial pressure, heart rate, or linear heart rate variability were detected between the groups. However, Ilex guayusa tea consumption (DG group) prevented the reduction in vascular sympathetic modulation, as indicated by lower LF-SAP and VAR-SAP, as seen in the D group. In fact, previous research, including our own, has consistently shown diminished vascular sympathetic modulation in STZ-induced diabetic rats [9,10,11]. Decreased autonomic modulation has long been recognized as an early marker of autonomic neuropathy in diabetes, often preceding overt symptoms or other manifestations of neuropathy [21]. Pathophysiologically, it is associated with impaired parasympathetic regulation by the vagus nerve, the longest autonomic nerve. This impairment occurs in a length-dependent manner, similar to the damage observed in peripheral somatic nerves affected by diabetic sensorimotor polyneuropathy [22]. Remarkably, this reduction, also considered a poor prognostic marker in this animal model of diabetes, was inversely correlated with glycemia. The significant inverse correlation between VAR-SAP and blood glucose levels observed in this study reinforces the notion that the metabolic derangements of diabetes are closely linked to autonomic dysfunction and cardiovascular impairment, characterized by the uncoupling of heart rate and blood pressure. However, these findings do not rule out the potential involvement of other central or peripheral control mechanisms in the pathophysiology of diabetes. Notably, Fiorino et al. [9] observed that green tea consumption also prevents VAR-SAP and LF-SAP reduction while improving glycemic control.

In addition, the STZ-induced diabetic rats treated with guayusa tea presented higher alpha 1, a non-linear analysis of HRV that belongs to detrended fluctuation analysis (DFA). DFA is a random-walk analytical method designed for analyzing nonstationary time-series data, first introduced by Peng et al. in 1995 [23]. In healthy individuals, HRV exhibits long-range power-law correlations, a fractal property resulting from the complex control mechanisms of heart rate regulation [24]. This regulation is mediated by the dynamic balance between sympathetic and parasympathetic nervous system activity, which influences both sinus node automaticity and atrioventricular node conduction. In the context of HRV analysis, DFA is commonly used to assess the autocorrelation function associated with heart rate regulation [23]. The scaling exponents derived from DFA offer insights into the system’s functionality. An exponent close to 1 suggests 1/f fluctuations, indicative of healthy and adaptive heart rate regulation. In contrast, a reduction in the scaling exponent reflects increased randomness, signaling pathological heart rate dysregulation and an impaired ability to respond to external disturbances. For example, Mizobuchi et al. [25] observed that patients with heart failure with a preserved ejection fraction presented an alpha 1 that was 27% lower than control subjects. Our group has also demonstrated that obese/diabetic mice (ob/ob) present a reduction of 43% in alpha 1 when compared to control lean mice [26].

Despite the high antioxidant levels of Ilex guayusa, no differences were observed between the groups regarding cardiac and brain oxidative stress. Perchance, a longer study period involving Ilex guayusa tea and an experimental diabetes model could reveal more significant changes from the perspective of oxidative stress.

Although this study offers novel insights into the potential benefits of Ilex guayusa tea on autonomic and metabolic parameters in diabetic rats, it has several limitations. First, the absence of a healthy, nondiabetic control group may restrict the generalizability of the findings. While published baseline data for nondiabetic animals were referenced to provide context, including such a group would have offered a more robust comparison. Second, the chemical composition of the Ilex guayusa extracts was not fully characterized to identify specific bioactive compounds, limiting the ability to link individual phytochemicals to the observed effects. Third, while the infusion method was chosen for its translational relevance, the decoction showed greater in vitro antioxidant activity and phenolic/flavonoid content, which may have influenced the outcomes. Lastly, histopathological analyses were not conducted, as tissue samples were prioritized for oxidative profile assessments. Future studies should address these gaps by including euglycemic groups, performing detailed phytochemical analyses, and integrating histopathological evaluations to enhance the interpretation and applicability of the findings.

These findings suggest that Ilex guayusa tea holds promise as an adjuvant therapy for diabetes mellitus, improving glycemic control and mitigating autonomic dysfunction. Further human studies are needed to optimize its use and evaluate its synergistic potential with pharmacological treatments.

## 4. Materials and Methods

### 4.1. Preparation of Aqueous Extracts of Ilex Guayusa

A decoction and an infusion were prepared as aqueous extracts using air-dried, mechanically milled aerial parts of Ilex guayusa tea (Runa, Quito, Ecuador) (5% *w*/*v*) according to the Argentine Pharmacopeia (Codex Medicamentario Argentino, 1979). For the decoction, the plant material was boiled in water at 100 °C for 20 min. The infusion involved steeping the plant material in boiling water for 20 min. Both preparations were filtered through paper filters and stored at −20 °C until further use.

### 4.2. Determination of Total Polyphenolic Content

The total polyphenolic content of the extracts was analyzed using the Folin–Ciocalteu method [27,28]. Aliquots (50 μL) of the infusions and decoctions were diluted to 0.5 mL with distilled water, followed by the addition of 0.25 mL of Folin–Ciocalteu reagent and 1.25 mL of 20% (*w*/*v*) sodium carbonate solution. After vortexing, the mixtures were incubated for 40 min, and absorbance was measured at 725 nm against a blank. A calibration curve was constructed using gallic acid at various concentrations as the standard. The content of total polyphenols is expressed as mg of gallic acid per g of dry plant material.

### 4.3. Determination of Total Flavonoid Content

The total flavonoid content was assessed using the aluminum chloride method [29]. The reaction mixture (1.25 mL) included 50 μL of aqueous extract, 450 μL of distilled water, 500 μL of aluminum chloride solution (1.2% *w*/*v*), and 250 μL of potassium acetate solution (120 mM). The mixture was incubated at room temperature for 30 min, and absorbance was measured at 415 nm. A calibration curve was constructed with quercetin as the standard at concentrations of 0, 11, 22, 33, and 44 μM. The results were expressed as milligrams of quercetin per gram of dry plant material.

### 4.4. Total Reactive Antioxidant Potential (TRAP)

TRAP was determined by chemiluminescence using a Luminoskan V 1.2-0 liquid scintillation counter. The reaction medium comprised 20 mM 2,2′-azobis(2-amidinopropane) (ABAP) and 40 mM luminol. Calibration was performed using trolox (0.25–0.50 mM), a vitamin E analog. Aliquots of 10 μL of infusion (1:20 dilution) and 5 μL of decoction (1:20 dilution) were analyzed, corresponding to total polyphenol contents of 0.98 mg and 0.64 mg gallic acid, respectively. Antioxidant capacity was calculated by comparing the induction times after adding trolox, infusion, or decoction, expressed as trolox equivalents [30].

### 4.5. Scavenging of ABTS Radical Cations

The ability to scavenge ABTS radical cations was evaluated in a reaction mixture containing 0.36 mM ABTS, 18 mM ABAP, and 100 mM phosphate buffer (pH 7.4). After a 45 min incubation at 45 °C, 10 μL of infusion or 5 μL of decoction was added to 3 mL of the solution, corresponding to 19.66 mg and 12.17 mg gallic acid, respectively. Absorbance was measured at 734 nm at a fixed time (3–4 min). A calibration curve using ascorbic acid (0, 4.75, 9.50, and 19.00 μM) was used, and the results were expressed as mmol ascorbic acid per gram of dry material [31].

### 4.6. Scavenging of DPPH Radicals

The scavenging activity against 2,2-diphenyl-2-picrylhydrazyl (DPPH) radicals was evaluated by measuring the reduction in absorbance at 515 nm over a fixed time of 10 min. The assay involved adding aliquots of the decoction or infusion to 3 mL of a DPPH solution (prepared by dissolving 2.5 mg of DPPH in 100 mL of methanol). Specifically, 10 mL of infusion and 5 mL of decoction were used, corresponding to total polyphenol contents of 19.66 mg and 12.17 mg gallic acid, respectively. A calibration curve was generated using ascorbic acid at concentrations of 0, 4.75, 9.50, and 19.00 μM. The results were expressed as millimoles of ascorbic acid equivalent per gram of dry plant material. This method provides an effective means of quantifying the antioxidant capacity of the extracts by determining their ability to neutralize stable DPPH radicals [32].

### 4.7. Reducing Power (RP) Assay

The reducing power of the extracts was determined using a spectrophotometric method. For the assay, 25 µL of 1:10-diluted infusion or decoction was combined with 175 µL of phosphate buffer (200 mM, pH 6.6) and 100 µL of potassium ferricyanide (30 mM). The mixture was incubated at 50 °C for 20 min, after which 100 µL of trichloroacetic acid (600 mM) was added. The reaction mixture was centrifuged at 3000 rpm for 10 min to separate layers. Next, 125 µL of the upper layer was mixed with 125 µL of distilled water and 1000 µL of ferric chloride (6 mM). The absorbance of the final solution was measured at 700 nm. A calibration curve was constructed using trolox at concentrations of 0, 6.25, 12.5, 25.0, and 37.5 µM. The reducing power of the extracts was expressed in terms of millimoles of trolox equivalents per gram of dry plant material. This assay evaluates the electron-donating capacity of the samples, reflecting their ability to reduce ferric ions to ferrous form, a key indicator of antioxidant potential [33].

### 4.8. Animal Studies

Experiments were performed using 13 female Wistar rats (10 weeks old) obtained from the Animal Facility of Nove de Julho University, Brazil. The rats received standard laboratory chow (Nuvital, Colombo, Brazil) and water/tea ad libitum. The animals were housed in individual cages in a temperature-controlled room (22 °C) with a 12 h dark–light cycle. Two experimental groups were used in this study: diabetic (D, *n* = 7), and diabetic treated with Ilex guayusa (DG, *n* = 6). All rats were similarly treated with regard to daily manipulation. All procedures and protocols were carried out in accordance with the ARRIVE guidelines and were approved by the Universidade Nove de Julho Ethics Committee (approval code: an0010.2016, approval date: 25 May 2016).

### 4.9. Diabetes Induction

Diabetes mellitus (DM) was induced by a single intravenous (IV) injection of streptozotocin (STZ, 50 mg/kg, Sigma Chemical Company, St. Louis, MO, USA) into the tail vein, following the method described by Rerup [34]. STZ was dissolved in citrate buffer (0.01 M, pH 4.5) and injected approximately 5 min after preparation. The animals were fasted for 6 h prior to induction.

### 4.10. Treatment with Ilex Guayusa

Treatment with Ilex guayusa began 7 days after diabetes induction. The preparation was made fresh daily by adding 3.0 g of Ilex guayusa (RUNA, Quito, Ecuador) to 1000 mL of boiling water (90 °C) for 20 min. The solution was steeped for 15 min, filtered, cooled to room temperature, and dispensed into thoroughly cleaned drinking bottles. This preparation was administered ad libitum for 24 h and repeated daily for 21 days. The D group received tap water for the same period of time.

### 4.11. Blood Glucose and Triglyceride Concentrations

Blood glucose concentrations were measured (Accucheck Roche) after 4 h fasting once per week. Blood triglyceride concentrations were measured (Accutrend, Roche) after 4 h fasting on the week of the protocol.

### 4.12. Hemodynamic Measurements

On the last day of the protocol, a catheter filled with 0.06 mL of saline was implanted in anesthetized rats (80 mg/kg ketamine and 10 mg/kg xylazine) into the carotid artery (PE-10) for direct measurements of AP. Rats receiving food and water ad libitum were studied 1 day after catheter placement; they remained conscious and were allowed to move freely during the experiments. An arterial cannula was connected to a transducer (Arterial Pressure XDCR, Kent© Scientific, Torrington, CT, USA), and AP signals were recorded over a 20 min period by a microcomputer equipped with an analog-to-digital converter board (Windaq, 2 kHz sampling frequency; Dataq Instruments, Inc., Akron, OH, USA). The recorded data were analyzed on a beat-to-beat basis to quantify changes in mean arterial pressure (MAP) and heart rate (HR). It is important to note that for all hemodynamic measurements and heart rate variability analyses, one group had *n* = 6 rats because one rat from the D group had its cannula occluded.

### 4.13. Heart Rate Variability (HRV) and Systolic Arterial Pressure Variability (SAPV)

The pulse interval signals used for the HRV and SAPV analyses were obtained as described above. Prior to HRV calculation, the recordings were manually reviewed and corrected for ectopic beats, arrhythmias, noise, and trends using the Windaq software (Version 2.19). Pulse interval detection was then performed within the Windaq software, followed by manual editing to ensure accurate identification of all pulse intervals. The processed data were saved as a Lotus file, compatible with MS Excel. This file was subsequently opened in Excel, where cumulative pulse interval values were converted into individual pulse interval series. This process allowed for determining the intervals between successive pulse intervals and the instantaneous heart rate values for each cardiac cycle.

*Linear analyses*: Time-domain variables included the root mean square of successive differences (RMSSD) and the standard deviation of pulse intervals (SD-PI). The power spectral density was calculated using the fast Fourier transform. The spectral power in the low-frequency (LF: 0.20–0.75 Hz) and high-frequency (HF: 0.75–4.0 Hz) bands was determined by integrating the power spectrum density over each frequency range using a custom MATLAB routine (MATLAB 6.0, MathWorks) [35]. In the context of SAPV, two variables were analyzed, VAR-SAP and LF-SAP. VAR-SAP represents the total variance in systolic arterial pressure fluctuations over a given period. It reflects the overall magnitude of blood pressure variability. LF-SAP is the power of SAP variability in the low-frequency (LF) band. It reflects the slow oscillations in blood pressure that are primarily mediated by sympathetic modulation of vascular tone. Increased VAR-SAP and LF-SAP may indicate heightened sympathetic activity.

*Non-linear analyses*: Poincaré plot analysis—the Poincaré plot provides a graphical representation of heart rate variability (HRV) by plotting each pulse interval (PI) against the preceding one. Scattergrams of successive PI intervals were generated for the entire recording period. Two standard deviations were calculated: SD1, reflecting short-term beat-to-beat variability and parasympathetic activity, and SD2, representing long-term HRV and overall variability [36].

*Detrended Fluctuation Analysis (DFA)*: The DFA method was employed to evaluate the fractal scaling properties of short- and intermediate-term PI interval time series. Root mean square fluctuations of integrated and detrended time series were calculated for different observation windows and plotted on a log–log scale. Alpha 1 is a scaling exponent that quantifies the short-term correlation properties of a time series. It is calculated over smaller time scales and provides insight into the fractal-like structure of heart rate dynamics. Alpha 2 reflects the long-term correlation properties of the signal over larger time scales. It quantifies the degree of self-similarity or long-range correlation in the HRV signal over these extended time periods. A detailed methodology is available in previous studies [37].

*Approximate Entropy (ApEn) and Sample Entropy (SampEn)*: Approximate entropy, introduced by Pincus in 1991, quantifies the regularity and complexity of time-series data, providing insights into signal abnormality and irregularity. Higher ApEn values indicate greater complexity. Similarly, SampEn was used to assess the complexity of heart rate signals under different conditions, measuring the likelihood that patterns close to each other remain similar in subsequent comparisons [38].

### 4.14. Oxidative Stress Profile on Brain and Cardiac Tissue

After the hemodynamics measurements, the animals were euthanized by decapitation, and tissues were harvested. Brain and left-ventricular cardiac tissues were minced into small pieces and homogenized in an ice-cold buffer using an ultra-Turrax blender (1 g of tissue per 5 mL of 150 mM KCl and 20 mM sodium phosphate buffer, pH 7.4). The homogenate was centrifuged at 600 g for 10 min at −26 °C.

### 4.15. NADPH Oxidase

The activity of NADPH oxidase enzyme was determined in the homogenate of heart and brain tissues. It was evaluated by the production of superoxide determined by evaluating superoxide production using a plate reader. The assay employed a 50 mM phosphate buffer containing 2 mM EDTA, 150 mM sucrose, 1.3 mM NADPH, and 10 μL of tissue sample. The results were expressed as μmol of superoxide per mg of protein.

### 4.16. Antioxidant Enzyme Activities

Superoxide dismutase (SOD) activity was quantified by measuring the inhibition of the reaction between superoxide anions (O_2_•⁻) and pyrogallol, with the results expressed as units of SOD per mg of protein. Catalase (CAT) activity was determined by monitoring the reduction in H_2_O_2_ absorbance at 240 nm, expressed as μmol H_2_O_2_ reduced per minute per mg of protein [39].

### 4.17. Membrane Lipoperoxidation by Thiobarbituric Acid Reactive Substances

For the TBARS assay, 10% trichloroacetic acid (*w*/*v*) was added to the tissue homogenates to precipitate proteins and acidify samples [34]. Following centrifugation at 3000× *g* for 3 min, the protein-free supernatant was mixed with 0.67% thiobarbituric acid (*w*/*v*) and heated in a water bath at 100 °C for 15 min. Absorbance was measured at 535 nm using a spectrophotometer. Malondialdehyde (MDA) was used as a standard, and the results were reported as μmol MDA per mg of protein [40].

### 4.18. Determination of Protein Oxidation Using Carbonyls Assay

Tissue samples were incubated with 10 mM 2,4-dinitrophenylhydrazine (DNPH) in 2.5 M HCl for 1 h at room temperature in the dark, with vortexing every 15 min. Proteins were precipitated by adding 20% trichloroacetic acid (*w*/*v*) and incubating the solution on ice for 10 min before centrifugation at 1000× *g* for 5 min. The pellet was washed three times with a 1:1 (*v*/*v*) ethanol–ethyl acetate solution. The final protein pellet was dissolved in 6 M guanidine hydrochloride and incubated at 37 °C for 10 min, and its absorbance was measured at 360 nm [41].

### 4.19. Statistical Analysis

Data are expressed as mean ± SEM. The Levene test was used to evaluate data homogeneity. Student’s *t*-test and two-way ANOVA for repeated measures analysis of variance followed by the Tukey test were used to compare groups. Cook’s distance (Di) test was used to determine potential outliers in the correlation analysis. Di > 1 was considered an outlier. Student’s *t*-test and ANOVA analysis were performed using Graphpad Prism (10.2.2), and Cook’s distance test was performed using R (4.0.3). Significance level was established at *p* ≤ 0.05.

## Figures and Tables

**Table 1:** Total antioxidant activity of Ilex guayusa, evaluated via TRAP, ABTS, DPPH, and RP assays.

Antioxidant Activity Assay	Infusion	Decoction	*p*
TRAP (μmol trolox per g dry material)	30.24 ± 5.10	49.64 ± 7.29	0.06
ABTS (μmol ascorbic acid per g dry material)	144.27 ± 20.46	324.72 ± 23.97	0.0002
DPPH (μmol ascorbic acid per g dry material)	85.07 ± 0.88	183.84 ± 3.66	>0.0001
RP (μmol trolox per g dry material)	361.17 ± 68.66	778.89 ± 57.86	0.0009

Values are expressed as mean ± standard error of mean. Results are mean values of three determinations.

**Table 2:** Phytochemical analysis of aqueous extracts of Ilex guayusa.

Phytochemical Analysis	Infusion	Decoction	*p*
Total polyphenolic content (mg gallic acid per g of dry material)	170.33 ± 12.20	322.96 ± 9.91	>0.0001
Total flavonoid content (mg quercetin per g of dry material)	5.49 ± 0.39	11.43 ± 0.47	>0.0001

Values are expressed as mean ± standard error of mean. Results are mean values of three determinations.

**Table 3:** Hemodynamic and autonomic parameters of the studied groups (D: *n* = 6 and DG: *n* = 6).

	D	DG	*p*
*Hemodynamic*			
Mean Arterial Pressure (mmHg)	103 ± 0.3	110 ± 3.4	0.33
Heart Rate (bpm)	295 ± 14	311 ± 9	0.38
*Linear HRV*			
SD (ms)	5.48 ± 1.16	7.67 ± 1.11	0.20
Variance (ms)	36.94 ± 14.56	64.98 ± 20.39	0.29
RMSSD (ms^2^)	7.84 ± 1.77	9.30 ± 0.55	0.45
VLF abs (ms^2^)	4.92 ± 1.88	13.41 ± 6.97	0.22
LF abs (ms^2^)	1.98 ± 0.91	3.24 ± 0.70	0.47
HF abs (ms^2^)	20.34 ± 8.96	24.66 ± 2.81	0.68
VLF %	20.33 ± 6.70	26.83 ± 6.74	0.39
LF %	7.16 ± 0.74	7.50 ± 0.71	0.87
HF %	72.50 ± 6.37	66.00 ± 6.46	0.40
LF (nu)	9.33 ± 1.05	10.33 ± 1.05	0.61
HF (nu)	90.66 ± 1.05	89.66 ± 1.05	0.61
LF/HF	0.10 ± 0.01	0.12 ± 0.01	0.66
*Non-Linear HRV*			
SD1 (ms)	5.5 ± 1.2	6.6 ± 0.4	0.45
SD2 (ms)	5.3 ± 1.1	8.4 ± 1.7	0.17
alpha 1	0.30 ± 0.02	0.40 ± 0.03	0.01
alpha 2	0.88 ± 0.01	0.98 ± 0.08	0.47
SampEn	2.17 ± 0.19	1.69 ± 0.1	0.05
ApEn	1.34 ± 0.07	1.48 ± 0.04	0.15
*Baroreflex Sensitivity*			
Alpha Index (ms/mmHg)	1.12 ± 0.30	1.97 ± 0.55	0.67

Values are expressed as mean ± standard error of mean. RMSSD, root mean square of the successive differences; LF, low-frequency band; HF, high-frequency band; Poincaré plot (SD1 and SD2); detrended fluctuation analysis (alpha 1 and alpha 2); sample entropy (SampEn); and approximate entropy (ApEn).

**Table 4:** Oxidative stress in the brain and cardiac tissues of the studied groups (D: *n* = 7 and DG: *n* = 6).

	Heart			Brain		
	D	DG	*p*	D	DG	*p*
CAT (nmol/mg protein)	2.35 ± 0.18	2.02 ± 0.21	0.52	0.24 ± 0.02	0.30 ± 0.02	0.09
SOD (USOD/mg protein)	14.86 ± 0.58	13.96 ± 0.53	0.30	10.47 ± 0.32	10.76 ± 0.46	0.61
TBARS (μmoles/mg protein)	2.33 ± 0.16	2.24 ± 0.18	0.71	3.16 ± 0.21	3.05 ± 0.16	0.69
CARB (nmol/mg protein)	3.71 ± 0.18	3.33 ± 0.12	0.13	3.20 ± 0.35	3.53 ± 0.29	0.50
NADPH Oxidase (nmol/mg protein)	0.192 ± 0.015	0.183 ± 0.009	0.63	0.040 ± 0.004	0.037 ± 0.004	0.60

Values are expressed as mean ± standard error of mean.

## Data Availability

The original contributions presented in this study are included in the article. Further inquiries can be directed to the corresponding authors.
